# P2X7 receptors exert a permissive effect on the activation of presynaptic AMPA receptors in rat trigeminal caudal nucleus glutamatergic nerve terminals

**DOI:** 10.1186/s10194-020-01153-y

**Published:** 2020-07-02

**Authors:** Diego Currò, Pierluigi Navarra, Irene Samengo, Maria Martire

**Affiliations:** Institute of Pharmacology, School of Medicine, Università Cattolica del Sacro Cuore, Largo Francesco Vito, 1, 00168 Rome, Italy

**Keywords:** P2X7 receptor, AMPA receptors, Trigeminal caudal nucleus, Synaptosomes, [^3^H]D-aspartic acid release, Central sensitization, Nociceptive circuit

## Abstract

**Background:**

Purine receptors play roles in peripheral and central sensitization and are associated with migraine headache. We investigated the possibility that ATP plays a permissive role in the activation of AMPA receptors thus inducing Glu release from nerve terminals isolated from the rat trigeminal caudal nucleus (TCN).

**Methods:**

Nerve endings isolated from the rat TCN were loaded with [^3^H]D-aspartic acid ([^3^H]D-ASP), layered into thermostated superfusion chambers, and perfused continuously with physiological medium, alone or with various test drugs. Radioactivity was measured to assess [^3^H]D-ASP release under different experimental conditions.

**Results:**

Synaptosomal [^3^H]D-ASP spontaneous release was stimulated by ATP and to an even greater extent by the ATP analogue benzoylbenzoylATP (BzATP). The stimulation of [^3^H]D-ASP basal release by the purinergic agonists was prevented by the selective P2X7 receptor antagonist A438079. AMPA had no effect on basal [^3^H]D-ASP release, but the release observed when synaptosomes were exposed to AMPA plus a purinoceptor agonist exceeded that observed with ATP or BzATP alone. The selective AMPA receptor antagonist NBQX blocked this “excess” release. Co-exposure to AMPA and BzATP, each at a concentration with no release-stimulating effects, evoked a significant increase in [^3^H]D-ASP basal release, which was prevented by exposure to a selective AMPA antagonist.

**Conclusions:**

P2X7 receptors expressed on glutamatergic nerve terminals in the rat TCN can mediate Glu release directly and indirectly by facilitating the activation of presynaptic AMPA receptors. The high level of glial ATP that occurs during chronic pain states can promote widespread release of Glu as well as can increase the function of AMPA receptors. In this manner, ATP contributes to the AMPA receptor activation involved in the onset and maintenance of the central sensitization associated with chronic pain.

## Background

ATP is known to be an important mediator of signal transmission through the spinal nociceptive circuits [1]. Its release by neurons and glial cells of these circuits impacts the activity of nearby cells by binding ligand-gated cation channels (P2X) or metabotropic (P2Y) receptors [2]. ATP interaction with P2X7 receptors elicits a dual-mode response, consisting of the rapid opening of a channel permeable to small cations (within milliseconds of binding) followed by the opening of a larger pore (seconds after ATP binding) through which molecules with a mass of up to 900 Da can flow. The second phase of the response appears to be mediated by the recruitment of additional pore-forming proteins, usually pannexin-1 (PANX1) but also connexins [3].

P2X7 receptors are widely expressed by several cell types involved in pain transmission, including the neurons, microglia, satellite glial cells, and astrocytes located in the dorsal root ganglia, the trigeminal ganglia, and in the dorsal horn of the spinal cord [4]. P2X7-knockout mice display a lack of hypersensitivity to mechanical and thermal stimuli [5], and P2X7-specific antagonists have consistently been found to produce protective effects in animal models of inflammatory and neuropathic pain [6]. In addition, selective centrally penetrant P2X7 antagonists are emerging as promising therapeutic agents, since the density of P2X7 receptors in healthy tissue is quite low [7]. ATP interacts with the neurotransmitters GABA and glutamate (Glu), and it has been shown to facilitate glutamatergic transmission in the spinal and medullary dorsal horn areas [8–10]. P2X7 receptors are found on excitatory nerve terminals in different areas of the CNS, including the medulla oblongata, spinal cord and nodose ganglia [11]. In the rat brain, P2X7 receptors are co-localized in nerve terminals with the vesicular Glu transporters vGLUT1 and vGLUT2 [12], and they have been shown to trigger Glu release by astrocytes [13] as well as neurons [14].

In this study, we examined interactions between P2X7 receptors and AMPA receptors co-expressed on primary afferents that from trigeminal ganglion (TG) project into the trigeminal caudal nucleus (TCN) of the rat brain. The TCN receives sensory input from the face and the head, including primary afferent Aδ- and C-fibers from the meningeal vessels. It is the first central relay station in the orofacial and head pain pathway. It contains second-order neurons whose nerve endings project into higher nerve centers and excitatory and inhibitory interneurons [15]. Nerve endings can be isolated from the rat TCN tissues that are presumably the glutamatergic terminals of both primary afferents and excitatory interneurons present in the TCN.

The nucleus caudalis of the trigeminal spinal tract is widely acknowledged to play an integral role in the brain stem processing of orofacial and head nociceptive signals, including those involved in migraine headache [16]. ATP and its catabolites are involved in various aspects of the initiation and transmission of migraine pain, including vasomotor mechanisms, cortical spreading depression, and fast transmission in neurons and glial cells [17]. The contributions of purinergic signaling to the transmission of migraine pain involves the activation of both P2X and P2Y receptors. Among P2X receptors, P2X3 receptors are almost exclusively expressed in nociceptive neurons, suggesting that they are involved specifically in the transmission of pain signals [18, 19]. P2X3 receptors interact with calcitonin gene-related peptide (CGRP), a central player in migraine pathogenesis [20], to trigger and amplify migraine pain. The trigeminal sensory neurons that innervate the meninges usually contain CGRP and express P2X3 receptors in their peripheral and central terminals as well [21]. The activation of P2X3 receptors facilitates the release of CGRP within the dura mater, thus promoting the initiation of inflammatory processes, and in the trigeminal nuclei of the brainstem [22]. CGRP causes sensitization of the nociceptive P2X3 receptors in the TG [23]. In addition, CGRP increases the levels of the pronociceptive nucleotides ATP and ADP in various parts of the trigeminal nociceptive system and enhances the responses induced by low concentrations of ATP [24]. While P2X3 receptors are involved in the initiation and transmission of a migraine attack, the metabotropic P2Y receptors seems to play modulatory roles in migraine pathophysiology [25]. The activation of P2Y13 metabotropic receptors causes contraction of the middle meningeal artery in vitro, reduces the dural artery dilation following periarterial electrical stimulation in vivo, and decreases CGRP release from both the dura and the trigeminal ganglion in situ. These are all opposite effects to those induced by P2X3 receptor activation, which include CGRP release and middle meningeal artery dilation [25]. P2X7 receptors also seem to play roles in the onset of migraine-associated neuroinflammatory events in the brain and meninges. Notably, migraine aura-related cortical spreading depression is associated with the opening of PANX1 channels, which are tightly coupled to P2X7 receptors, suggesting that a massive release of ATP occurs during a migraine attack [26, 27]. Furthermore, P2X7 receptors, that are ligand-gated ion channels that conduct Na^+^ and Ca^2+^ [28], can trigger the exocytotic release of Glu, a features which is also compatible with their putative role in determining the susceptibility to the spreading depolarization.

In this study, we investigated the possibility that ATP co-released with Glu from rat TCN nerve terminals exerts a permissive effect on the activation of AMPA receptors that mediate Glu release. To this end, we assessed the release of pre-loaded [^3^H]D-aspartate [[^3^H]D-ASP) from superfused rat trigeminal synaptosomes. We found that the activation of ATP receptors belonging to the P2X7 subtype not only elicits Glu release, but also facilitates the activation of release-enhancing AMPA receptors co-existing with P2X7 receptors on TCN glutamatergic terminals.

## Methods

### Animals and synaptosomes preparation

Adult male Wistar rats (weighing 200–250 g) (Animal Facility of Catholic University) were used in the study. Animal procedures were approved by the Ethics Committee of the Catholic University and were fully compliant with Italian (Ministry of Health guidelines, Legislative Decree No. 116/1992) and European (Directive No. 86/609/EEC) animal research legislation.

Animals were sacrificed, and the brain and upper portion of the spinal cord were rapidly removed and immersed in ice-cold medium (described in detail below). The cerebral cortex and cerebellum were discarded, and the tissue block containing the brainstem was sectioned at the level of the obex and 0.4 cm below the obex to isolate the brainstem segment containing the TCN [29]. The lateralmost portions (right and left) of this tissue block, which contained the TCN, were used to prepare crude synaptosomes, as previously described [30].

### Release experiments

The synaptosome pellet was resuspended in standard perfusion medium, a physiological medium containing (mmol/L) NaCl 125, KCl 3, MgSO_4_ 1.2, CaCl_2_ 1.2, NaH_2_PO4 1.0, NaHCO_3_ 22, and glucose 10 (oxygenated with 95% O_2_/5% CO_2_, pH 7.40)_._ The synaptosomes were then incubated in an atmosphere of 95% O_2_/5% CO_2_ for 15 min at 37 °C with 0.03 μM [^3^H]D-ASP. Exogenously loaded [^3^H]D-ASP is widely used in synaptosomal release experiments as a marker for endogenously formed Glu since the two substances are similarly released, via an exocytotic Ca^2+^-dependent process, in response to 12 mM [K^+^]_e_) [31]. Use of this mild depolarizing stimulus allows us to obtain a “clean” estimation of Ca^2+^-dependent Glu exocytosis, without the component mediated by the transporter reversal, which can occur when the extracellular Na^+^ level is reduced to compensate for the increase in K^+^ in the depolarizing medium [32].

Identical aliquots of the synaptosomal suspension were placed on 0.8 μm Millipore filters at the bottom of a set of parallel superfusion chambers, which were maintained at 37 °C [33]. The synaptosome suspension was then washed with 3 × 10 ml of standard medium at 37 °C under moderate vacuum filtration and superfused at a rate of 0.6 ml/min with standard medium aerated with 95% O_2_/5% CO_2_. After a 38-min equilibration period, the synaptosomes were perfused with standard medium with or without test substances or depolarizing medium (standard medium subjected to equimolar substitution of 12 mM NaCl with KCl) with or without test substances. Thereafter perfusion was continued with standard medium alone for different intervals, depending on the experimental protocol. Antagonists, when tested, were added to the medium 8 min before the agonists. The effect of lowering extracellular Mg^2+^ concentration was assessed in synaptosomes superfused with a medium containing 0.01 mmol/L MgSO_4_, starting 18 min before addition of the agonist. More detailed descriptions of experimental methods are provided in the figure legends.

Superfusate fractions were collected every 2 min, starting from min 38 of the pre-stimulation phase, and radioactivity was counted in each fraction as well as in the superfused synaptosomes themselves.

The amount of radioactivity found in a given superfusate fraction was expressed as a percentage of the total tritium present in the synaptosomes at the beginning of the respective collection period. These percentages were then plotted against perfusion time (min) to evaluate the time-course of [^3^H] D -ASP release under different conditions. To evaluate the effects of test drugs, we compared the integrated areas under the time/release curves (AUCs) obtained in the presence of the test drug and under control conditions (assessed in parallel). The results were expressed as percentage increases or decreases relative to control values.

### Statistical analyses

Reported data represent means ± S.E.M. of the given number of experiments (*n*).

Each experiment (i.e., *n* = 1) was carried out using tissues isolated from the TCNs of five rats. We put together and homogenized the five TCNs (thereby annulling individual differences between one animal and another) to produce a single sample large enough to be layered onto the bases of multiple perfusion chambers. The mean of values observed under identical experimental conditions in 3 perfusion chambers was calculated and represents 1 *n*. A total of 120 rats were used for the 24 experiments conducted.

Analysis of variance was performed by ANOVA followed by Dunnett’s multiple comparison test. *P* values < 0.05 were considered significant.

### Drugs

D-[2, 3-^3^H] -aspartic acid ([^3^H] D -ASP) (specific activity 11.30 Ci/mmol) was purchased from Perkin Elmer Life and Analytical Sciences (Boston, MA, USA). Dihydrokainate; 6-chloro-3,4-dihydro-3-(5-norbornene-2-yl)-2H-1,2,4-benzothiazidiazine-7-sulfonamide-1,1-dioxide (cyclothiazide); 2,3-dioxo-6-nitro-1,2,3,4-tetrahydrobenzo [f]quinoxaline-7-sulfonamide disodium salt (NBQX); adenosine 5′-triphosphate disodium salt (ATP); 2′-3′-O-(4-benzoylbenzoyl) adenosine 5′-triphosphate triethylammonium salt (BzATP); 3-[[5-(2,3-dichlorophenyl)-1H-tetrazol-1-yl] methyl] pyridine hydrochloride (A438079) were purchased from Tocris Cookson (Bristol, United Kingdom). (±)-α-Amino-3-hydroxy-5-methylisoxazole-4-propionic acid hydrobromide (AMPA) was purchased from Sigma–Aldrich (St. Louis, MO, USA). Initially, we prepared stock solutions of AMPA and stored them at − 20 °C until use, but their activity was found to decrease over time. The experiments reported below were therefore performed with AMPA solutions prepared from powder on the day of the experiment. When possible, drugs were dissolved in distilled water or in standard perfusion medium. Stock solutions of cyclothiazide (CTZ) were prepared in dimethyl sulfoxide and diluted at least 1:1000 in standard medium.

## Results

**Fig. 1:**
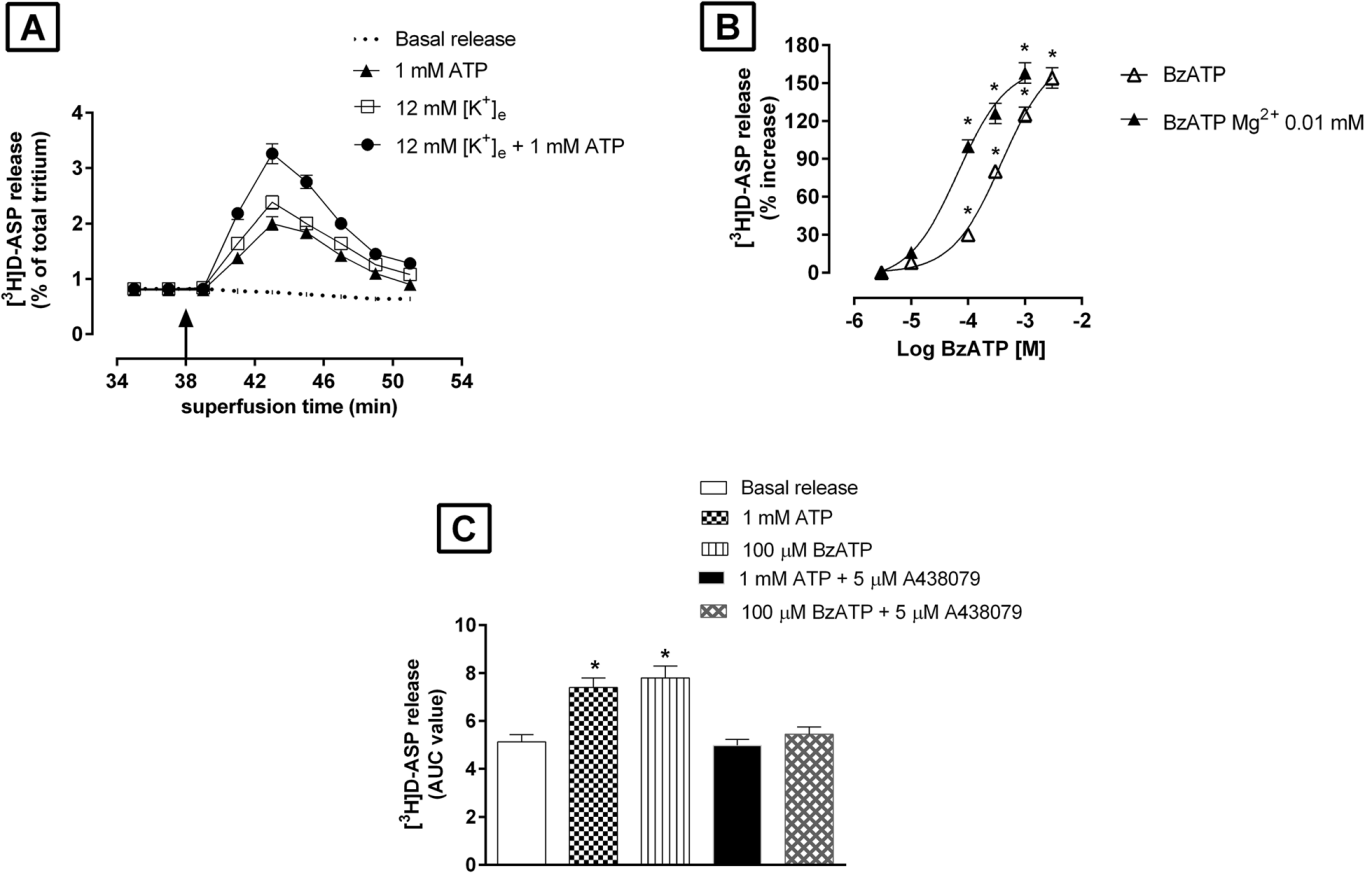
Effects of purinergic receptor agonists on basal and 12 mM [K^+^]_e_-evoked [^3^H]D-ASP release from rat TCN synaptosomes. **a** Effects of ATP on basal and [K^+^]_e_-evoked release of [^3^H]D-ASP. Synaptosomes prelabeled with [^3^H]D-ASP were superfused with standard medium at a flow rate of 0.6 ml/min. At the beginning of min 38 (arrow), the medium was replaced with standard medium containing ATP (1 mM) or depolarizing medium (12 mM [K^+^]_e_) plus ATP (1 mM) (arrow) and perfusion continued for 4 min (i.e., minute 38 through min 41). Radioactivity in each fraction has been expressed as a percentage of the total radioactivity content present in the tissue at the onset of the fraction collected. Each point represents the mean ± S.E.M. of 3–4 different experiments, each run in triplicate (3 superfusion chambers for each condition). Error bars are absent when the S.E.M. was smaller than the symbol used in the graph. **b** Log concentration-response curves for BzATP in evoking [^3^H]D-ASP efflux and its Mg^2+^ sensitivity. BzATP (0.01–3 mM) was present in the standard perfusion medium for 4 min (min 38 through min 41). To assess the Mg^2+^–sensitivity of the effects of BzATP, synaptosomes were perfused for 18 min (min 20 through min 37) with standard medium in which the Mg^2+^ concentration had been reduced from 1.2 mmol/L to 0.01 mmol/L and then with the same medium containing BzATP (min 38 through min 41). The effects of the BzATP were expressed as percentage increases (ratio of the AUC observed in the presence of BzATP to the AUC for the release observed in the absence of the agonist). Data are means ± S.E.M. of at least 3 experiments, each run in triplicate. *P < 0.05 vs basal release. **c** Effects of ATP and BzATP on basal [^3^H]D-ASP release and their antagonism by A438079. A438079 (5 μM) was added to the perfusion medium 8 min before the agonists. ATP (1 mM) or BzATP (100 μM) was present in the standard perfusion medium (with or without A438079) for 4 min (minutes 38 through 41). Results are expressed as areas under the release/time-curves (AUCs). Each bar represents the mean ± SEM of 3 different experiments, each run in triplicate. *P < 0.05 vs basal release

Synaptosomes isolated from rat TCN and preloaded with [^3^H]D-ASP were superfused with standard medium or with standard medium followed by depolarizing medium. Typical time-courses of basal tritium release and that evoked by depolarization (12 mM [K^+^]_e_) are illustrated in Fig. 1a. As shown in Fig. 1a, 1 mM ATP stimulated basal release of [^3^H]D-ASP from synaptosomes, as well as that triggered by 12 mM [K^+^]_e_. The highest concentration of ATP used (3 mM) increased by 85 ± 5% the basal [^3^H]D-ASP release.

The enhancement of basal release was more evident when synaptosomes were exposed to benzoylbenzoyl-ATP (BzATP), a nonselective P2X receptor agonist whose potency in activating P2X7 receptors (but not other P2X receptor subtypes) exceeds that of ATP by at least one order of magnitude [34]. At concentrations of 0.01–3 mM, the effects of BzATP were concentration-dependent (Fig. 1b) with an EC_50_ of 245 ± 12.50 μM (*n* = 3) and maximum enhancement of 152 ± 6.50%.

The activities of the various types of P2X receptors are known to be influenced by divalent cation concentrations [35]. We therefore assessed the effects of extracellular Mg^2+^ levels on BzATP-evoked tritium release from the TCN synaptosomes. As shown in Fig. 1b, the concentration-response curve displayed a clear shift to the left when the Mg^2+^ level of the medium was reduced from 1.2 mmol/L Mg^2+^ to 0.01 mmol/L Mg^2+^: in the low Mg^2+^ medium, the maximum effect was 148 ± 10% and EC_50_ was 24 ± 1.40 μM (*n* = 3).

As shown in Fig. 1c, the increases in [^3^H]D-ASP release induced by 1 mM ATP or by 100 μM BzATP were completely reversed by the selective P2X7-receptor antagonist A438079 (5 μM).

**Fig. 2:**
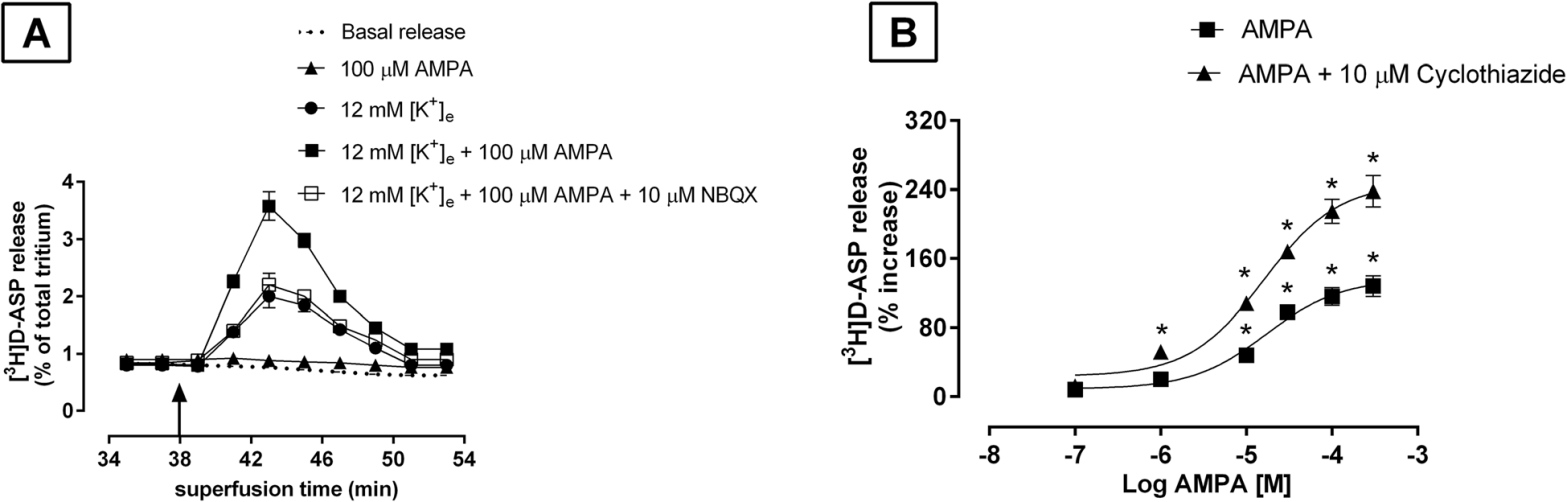
Effects of AMPA on basal and 12 mM [K^+^]_e_-evoked [^3^H]D-ASP release from rat TCN synaptosomes. **a** Effects of AMPA and antagonism by NBQX. Synaptosomes were prepared as previously described, and perfused for 37 min with standard medium. From min 38 through min 41, perfusion was carried out with standard medium containing 100 μM AMPA or depolarizing medium plus 100 μM AMPA. When used, the antagonist NBQX (10 μM) was added to the medium 8 min before AMPA. Each point represents the mean ± S.E.M. of 3–4 different experiments, each run in triplicate. **b** Cyclothiazide-sensitivity. The AMPA receptor desensitization inhibitor cyclothiazide (CTZ) (10 μM) was added concomitantly with AMPA (0.1–300 μM) to the depolarizing medium. The effects of the drugs on evoked release are expressed as percentage increases over values observed in the presence of the depolarizing stimulus alone. Data are means ± SEM of 3 experiments, each run in triplicate. *P < 0.05 vs [K^+^]_e_-evoked [^3^H]D-ASP release

Glutamatergic nerve terminals isolated from the rat TCN express autoreceptors for AMPA that can potentiate the [^3^H]D-ASP release induced by depolarization [10]. When synaptosomes were exposed in superfusion to 100 μM AMPA in medium containing 12 mM [K^+^]_e_, a significant increase of [K^+^]_e_-evoked tritium release was observed (124 ± 7%; *n* = 3) (Fig. 2a), although AMPA (100 μM) had no significant effect on basal [^3^H]D-ASP release (Fig. 2a). The 100 μM AMPA-induced potentiation of [K^+^]_e_-evoked [^3^H]D-ASP release was abolished by the selective AMPA antagonist NBQX (10 μM) (Fig. 2a), and it was significantly enhanced by cyclothiazide (CTZ) (10 μM), an agent capable of inhibiting the rapid desensitization of AMPA receptors.

The dose-response curve with the various concentrations of AMPA (0.1–300 μM) + 10 μM CTZ showed a maximum effect of 240 ± 15% and an EC_50_ of 16 ± 1.20 μM (*n* = 3) (Fig. 2b). Used alone at the concentrations tested, neither CTZ nor NBQX had any effect on [K^+^]_e_-evoked [^3^H]D-ASP release (data not shown).

**Fig. 3:**
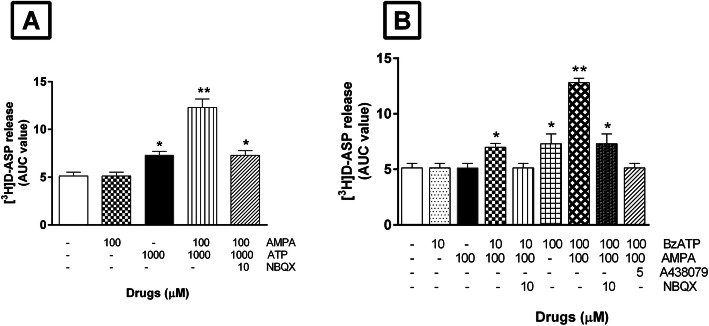
Effects of AMPA and ATP (or BzATP) alone or in combination on basal [^3^H]D-ASP release: **a** Effects of AMPA and ATP alone or in combination on basal [^3^H]D-ASP release and their antagonism by NBQX. After 38 min of perfusion with standard medium, synaptosomes were exposed for 4 min to standard medium containing AMPA (100 μM), ATP (1 mM), or both drugs together. When used, the antagonist NBQX (10 μM) was added to the medium 8 min before the agonists. All results are expressed as areas under the release/time curves (AUCs). Each bar represents the mean ± SEM of 3 different experiments, each run in triplicate. *P < 0.05 vs basal release. **P < 0.01 vs basal release. **b** Effects of BzATP and AMPA alone or in combination and their antagonism by A438079 and by NBQX. The effects of BzATP were tested at two concentrations (10 and 100 μM), each in the presence or absence of AMPA (100 μM). Exposure times to agonists and antagonists (A438079 and NBQX) are those described in panel A. Each bar represents the mean ± SEM of 3 different experiments, each run in triplicate. *P < 0.05 vs basal release. **P < 0.01 vs basal release

The findings reported above suggest that P2X7 receptors and AMPA receptors on glutamatergic terminals in the TCN might be interacting to regulate the latters’ release of Glu. To investigate this possibility, we measured [^3^H] D -ASP release from synaptosomes perfused with standard medium containing 100 μM AMPA, with or without 1 mM ATP. As shown in Fig. 3a, AMPA (100 μM) alone had no effect on basal release, but when it was added to standard medium containing 1 mM ATP, it appreciably potentiated the ATP-induced release of [^3^H]D-ASP, with an increase in basal release of 135 ± 8.50% (*n* = 3) vs the increase produced by 1 mM ATP alone (40 ± 2.50%; *n* = 4). The potentiating effect of AMPA was abolished by the presence of the AMPA antagonist NBQX (10 μM) (Fig. 3a).

Millimolar concentrations of ATP are reportedly necessary to produce functional effects mediated by P2X7 purinoceptors [34, 36]. Since such concentrations are likely to have multiple actions, our subsequent experiments were performed with BzATP, which is more potent than ATP in activating the P2X7 receptor subtype [34].

As shown in Fig. 3b, BzATP 10 μM alone had no effect on the basal release of [^3^H]D-ASP by synaptosomes, but a significant increase was observed when BzATP was used at a concentration of 100 μM (42 ± 8%; *n* = 3). When BzATP (100 μM) was applied with AMPA (100 μM), which had no effect on basal release when used alone, the increased release was significantly greater than that observed with BzATP alone (148 ± 9%; *n* = 3).

The potentiation of the BzATP-evoked release seen in the presence of AMPA was not observed when NBQX (10 μM) was present in the superfusion medium, indicating that AMPA receptors are likely to be involved in this potentiation. The marked increase in basal [^3^H]D-ASP release induced by BzATP plus AMPA suggests that activation of purinergic receptors located on glutamatergic nerve endings in the TCN may facilitate the exposure of AMPA receptors located on the same nerve terminals.

To shed light on the mechanism of the interaction between P2X7 and AMPA receptors, we investigated the possibility that [^3^H]D-ASP release would be elicited by concomitant exposure to BzATP and AMPA, each used at concentrations that were per se inactive. As shown in Fig. 3b, addition to the superfusion solution of 10 μM BzATP and 100 μM AMPA provoked significant enhancement of basal [^3^H]D-ASP efflux (35 ± 2%; *n* = 3), an effect that was abolished by the AMPA antagonist NBQX (10 μM) and also by the P2X7 receptor antagonist A438079 (5 μM) (Fig. 3b).

## Discussion

Glutamate plays major roles in the transmission and modulation of pain information in the trigeminal sensory system [37]. In addition, it is regarded as an important mediator of central sensitization that stems from changes in ionotropic NMDA and AMPA Glu receptors caused in part by the translocation of intracytoplasmic receptors to the membrane and in part by phosphorylation-mediated increase in receptor/ion channel function [38, 39]. Inhibition of spinal AMPA receptors has been shown to reverse the mechanical allodynia and the thermal and mechanical hypersensitivities associated with the development and maintenance of various types of pain [40, 41]. However, the therapeutic implications of these findings are limited in that blockade of AMPA receptors with competitive antagonists also affects basal peripheral sensitivity and causes sedation [41, 42].

Our findings show that purinergic P2X7 receptors are functionally linked with presynaptic AMPA receptors on glutamatergic axon terminals in the rat TCN. P2X7 receptors agonists were found to facilitate the activation of the AMPA receptors, suggesting that, in addition to provoking Glu release on its own, ATP can also regulate AMPA-mediated Glu release. The technique of up-down superfusion of synaptosomes used in this study has previously been exploited to identify and characterize release-regulating presynaptic receptors [33]. In our perfusion apparatus, a thin layer of synaptosomes is perfused under conditions that preclude indirect effects: the vertical movement of the perfusate rapidly removes compounds released by the nerve terminals before they can activate targets expressed by the releasing terminals themselves or by particles in the vicinity of the latter. These presynaptic targets thus remain virtually unbound by endogenous ligands and can be selectively activated by agents added to the superfusion medium.

In our experiments, ATP stimulated the release of preloaded [^3^H]D-ASP from superfused glutamatergic terminals in the rat TCN. On the basis of the results obtained, the pharmacological profile of the receptor that mediates the increase of ATP in basal [^3^H]D-ASP release from glutamatergic terminals in the TCN resembles that of the P2X7 subtype. First of all, the effects ATP were observed at millimolar concentrations [34, 36]. We can reasonably assume that in our superfusion system, where the medium is continually renewed, ATP catabolism does not significantly affect ATP concentrations. Second, both ATP and BzATP evoked [^3^H]D-ASP release, but the superior potency of the latter in this setting points strongly to the involvement of P2X7 receptors [34]. Last but not least, the response to ATP was abolished by the selective P2X7 antagonist A438079, and the response to BzATP was potentiated by the reduction of extracellular Mg^2+^ levels [35, 43].

Glutamatergic axon terminals in the rat brain express facilitatory AMPA autoreceptors capable of enhancing Glu release [44, 45]. In the rat TCN, AMPA receptors located on glutamatergic nerve endings have been shown to facilitate [^3^H]D-ASP release during terminal depolarization [10], and their activity can be modulated by CTZ, which inhibits the rapid desensitization typical of AMPA receptors inhibiting receptor endocytosis [46]. A dynamic equilibrium is normally maintained between the number of AMPA receptors expressed at the cell membrane and cytosolic compartments [47]. It has been proposed that AMPA receptors move from and to presynaptic membranes [48], as they reportedly do at the postsynaptic level. In the spinal cord, the largest AMPA receptor populations consist of the GluA1/GluA2 and GluA2/GluA3 heteromers [49]. Under basal conditions, excitatory neurons appear to express mostly the non–Ca^2+^-permeable GluA2 subunit [50, 51]. AMPA receptors containing the GluA2 subunits are continuously inserted into synapses, whereas synaptic activity is required for insertion of those containing the GluA1 subunit [52, 53]. The results of our experiments are compatible with the view that glutamatergic terminals in the rat TCN express CTZ-sensitive, GluA2-containing, heteromeric AMPA-preferring autoreceptors, which translocate constitutively into and out of presynaptic plasma membranes.

We found that: (i) AMPA is unable to evoke [^3^H]D-ASP release under basal conditions; (ii) the increase in spontaneous [^3^H]D-ASP release is triggered by ATP and to an even greater extent by BzATP; (iii) the [^3^H]D-ASP release evoked by co-application of purinoceptor agonists and AMPA exceeds that elicited by application of ATP or BzATP alone; and (iv) this “excess” release is mediated by AMPA-receptor activation. As noted above, the technique we used to monitor [^3^H] D -ASP release allows us to exclude the possibility of indirect effects. We can therefore reasonably conclude that activation of P2X7 receptors is exerting a permissive effect on the activation of AMPA receptors located on the same glutamatergic terminals. Importantly, this hypothesis is supported by the fact that co-application of AMPA and the P2X7 receptor agonist BzATP —each at a concentration unable to evoke [^3^H]D-ASP release—did indeed elicit significant release of [^3^H]D-ASP, which could be prevented by the selective NBQX AMPA antagonist.

Our results suggest that activation of P2X7 receptors permits Na^+^ and Ca^2+^ influx, depolarization of the nerve terminal, and stimulation of [^3^H]D-ASP release. Concentrations of the P2X7 agonist that have no effect on basal [^3^H]D-ASP release could nonetheless cause increases in cytosolic Ca^2+^ levels sufficient to permit activation of AMPA receptors by the following mechanism. The cytosolic proteins involved in AMPA receptor trafficking are Ca^2+^ sensors [54], which raises the possibility that Ca^2+^ ions flowing through P2X7 receptor-associated channels can transduce P2X7 receptor activation into an increased presence of AMPA receptors at the level of the synaptic plasma membranes. Consequently, P2X7-mediated increases in the number of AMPA receptors expressed on the presynaptic membrane would allow nerve terminal depolarization and the release of Glu.

It has been shown that ATP released from glial cells in response to the activation of α_1_-adrenoceptors reportedly increases the strength of glutamatergic synapses by promoting the phosphatidylinositol 3-kinase (PI3K)-dependent insertion of postsynaptic AMPA receptors [55]. Understanding the function of cerebral AMPA receptors and the mechanisms underlying their regulation is important for several reasons. AMPA receptor-mediated glutamatergic transmission plays pivotal roles in modulating the neuronal and synaptic activities in many brain regions. In addition, the auxiliary subunits that regulate AMPA-receptor trafficking in several brain areas have been proposed as valuable therapeutic targets [56]. A clearer understanding of the mechanisms regulating the AMPA receptors involved in transmitting pain signals in specific brain areas could shed valuable light on the properties of these receptors. Our findings show that presynaptic AMPA receptors can be activated by nerve terminal depolarization or by the activation of P2X7 receptors co-localized on those terminals. Most nerve terminals are capable of storing and releasing ATP (as a fast co-transmitter) together with a classical fast transmitter. The role of ATP as a co-transmitter varies considerably in different species and pathological conditions, but also within neuronal systems, depending on the transmitter it partners with and the receptors involved [57]. P2X7 receptors are activated by high concentrations of ATP. It is therefore conceivable that, during states of augmented neuronal activity capable of triggering more sustained ATP release, P2X7 receptors could activate AMPA receptors and facilitate the release of Glu [58, 59].

Some investigators have suggested that P2X7 receptors might be physiologically silent, due to the low extracellular concentrations of ATP, and their expression and/or activation is restricted to pathological states (e.g., inflammation, trauma, stress), characterized by metabolic distress or cellular damage favoring an ATP-rich extracellular milieu near the receptors [60, 61]. The high extracellular levels of ATP reached during such states could trigger widespread activation of P2X7 receptors, including those present in nearby astrocytes and microglia [61]. Since P2X7 receptor activation can also trigger Glu release from astrocytes [13], it is reasonable to hypothesize that ATP and Glu released by glial cells influence the neuronal release of Glu via their co-activation of presynaptic AMPA receptors. In this manner, P2X7 receptors would be capable of triggering Glu release from the nerve terminals and facilitating the transmission of nociceptive signals. P2X7 receptors have been proposed as central players in the synergic cross-talk that occurs between the neural network and nearby glial cells and serves to ensure the high-level synaptic transmission that reinforces pain signals [62]. P2X7 receptor-mediated neuronal-glial interaction could give rise to a form of plasticity involving multiple synapses: the high level of ATP release by glial cells activated during states of chronic neuropathic and/or neuroinflammatory pain would promote Glu release on a widespread scale as well as the function of specific AMPA receptors.

## Conclusion

The data presented here indicate that P2X7 and AMPA receptors located on glutamatergic nerve terminals in the TCN can interact to synergically regulate Glu release. The abundant release of glial ATP that occurs during chronic neuropathic/neuroinflammatory pain promotes a diffuse increase in extracellular Glu to pathological levels by directly stimulating the release of excitatory neurotransmitter and by facilitating the function of presynaptic AMPA receptors.

## Data Availability

The datasets analyzed for this study are available on reasonable request to the corresponding author.

## Abbreviations

AMPA: Aminomethylisoxazole propionic acid
ANOVA: Analysis of variance
ATP: Adenosine triphosphate
AUC: Area under the curve
BzATP: Benzoylbenzoyl ATP
D-ASP: D-aspartic acid
GABA: γ-Aminobutyric acid
NBQX: Nitro-tetrahydrobenzo [*f*]quinoxaline-7-sulfonamide
SEM: Standard error of the mean
TCN: Trigeminal caudal nucleus
